# Subkutaner Defibrillator (S-ICD)

**DOI:** 10.1007/s00059-025-05344-8

**Published:** 2025-10-07

**Authors:** Jürgen Kuschyk, Fabian Fastenrath, Katherine Sattler, Ibrahim Akin, Daniel Duerschmied, Boris Rudic

**Affiliations:** 1 I. Medizinische Klinik, Sektion für Invasive Kardiologie und Elektrophysiologie, Universitätsmedizin Mannheim, Mannheim, Deutschland; 2 Sektion für Invasive Kardiologie und Elektrophysiologie, I. Medizinische Klinik, Kardiologie, Hämostaseologie und Internistische Intensivmedizin, Universitätsmedizin Mannheim, Theodor-Kutzer-Ufer 1–3, 68167 Mannheim, Deutschland

**Keywords:** Defibrillatortherapie, Extrathorakales System, Patientenselektion, Implantation, Modular ATP/EMPOWER, Defibrillator treatment, Extrathoracic system, Patient selection, Implantation, Modular ATP/EMPOWER

## Abstract

Die Defibrillatortherapie muss leitlinienkonform erfolgen und zuverlässig vor dem plötzlichen Herztod schützen, während gleichzeitig alle Komplikationen minimiert werden. Der subkutane implantierbare Kardioverter-Defibrillator (S-ICD) vermeidet als komplett extrathorakales System intrakardiale Elektroden und reduziert damit prozedurale, elektrodenassoziierte Komplikationen und systemische Infektionen. Die Evidenz (> 5000 Teilnehmende in randomisierten Studien und Registern) demonstriert eine etwa 98 %ige Schockeffektivität und niedrige Komplikationsraten. Die PRAETORIAN-Studie belegt die Nichtunterlegenheit gegenüber dem transvenösen Defibrillator (TV-ICD); Langzeit- und Sekundäranalysen dokumentieren weniger schwerwiegende und insbesondere elektrodenassoziierte Komplikationen unter der S‑ICD-Therapie. Studienergebnisse aus UNTOUCHED, EFFORTLESS und PAS bestätigen Schockeffektivität und Sicherheitsprofil im klinischen Alltag; ATLAS zeigt bei jüngeren Patientinnen und Patienten signifikant weniger perioperative elektrodenassoziierte Komplikationen. Die Prozedur ist heute standardisiert („best practices“: präoperatives Vektorscreening, Zweischnitttechnik, inter-/submuskuläre Tasche, Dual-zone-Programmierung mit Morphologie‑/Hochpassfiltern). Bei fehlendem Bedarf an Brady-Pacing, kardialer Resynchronisationstherapie (CRT) oder antitachykardem Pacing (ATP) sollte der S‑ICD im klinischen Alltag eine regelhafte Option sein, insbesondere bei jüngeren, infektgefährdeten oder venös limitierten Betroffenen sowie bei hereditären Arrhythmiesyndromen.

**Video online:**

Die Online-Version dieses Beitrags (10.1007/s00059-025-05344-8) enthält Videos.

## Abstract (en)

Defibrillator treatment should adhere to the guidelines to effectively prevent sudden cardiac death while minimizing all complications. As an entirely extrathoracic system, the subcutaneous implantable cardioverter defibrillator (S-ICD) eliminates the need for intracardiac leads, thus reducing procedural and lead-related complications as well as systemic infections. The evidence across > 5000 participants in randomized trials and registries demonstrates approximately 98% shock conversion with low complication rates. The PRAETORIAN study demonstrated that the S‑ICD is not inferior to the transvenous ICD (TV-ICD), long-term and secondary analyses indicate fewer severe, particularly lead-related, complications with S‑ICD treatment. Studies such as UNTOUCHED, EFFORTLESS, and PAS confirm its shock effectiveness and safety profile in routine clinical care, while the ATLAS study reports significantly fewer perioperative lead-related complications in younger patients. The procedure has been streamlined and standardized, incorporating best practices such as preoperative vector screening, a two-incision technique, intermuscular/submuscular pocket creation, and dual-zone programming with morphology/high-pass filtering. In the absence of indications for bradypacing, cardiac resynchronization therapy (CRT) or antitachycardia pacing (ATP), the S‑ICD should be considered a routine option in the clinical routine, especially for younger patients, those prone to infections, those with limited venous access and individuals with hereditary arrhythmia syndromes.

## Einleitung

Der plötzliche Herztod („sudden cardiac death“, SCD) ist alles andere als „tot“ – er bleibt eine der häufigsten Ursachen kardiovaskulärer Mortalität [1–4]. Meist liegt eine maligne ventrikuläre Arrhythmie zugrunde, nicht selten als erste Manifestation einer strukturellen Herzerkrankung. Betroffen sind nicht nur Patienten mit fortgeschrittener Herzinsuffizienz oder Kardiomyopathien, sondern auch solche mit hereditären Arrhythmiesyndromen wie Long/Short-QT-, Brugada- oder katecholaminerges polymorphes ventrikuläres Tachykardiesyndrom (CPVT).

Die Implantation eines implantierbaren Kardioverter-Defibrillators (ICD) ist deshalb weiterhin der Goldstandard zur Sekundär- und – bei entsprechendem Risikoprofil – auch zur Primärprävention [5–7]. Klassische transvenöse Systeme bieten eine exzellente Schutzwirkung, sind jedoch mit spezifischen Komplikationen verbunden: Neben relevanten Elektrodendefekten über die Zeit (25–40 % in 10 Jahren) und venösen Obstruktionen sind systemische Infektionen inkl. Sondenendokarditiden gefürchtet, die eine komplette Systemextraktion erfordern und mit einer erheblichen Morbidität und Mortalität einhergehen [8–11]. Auch eine relevante Beeinträchtigung der Trikuspidalklappenfunktion kann die Prognose beeinflussen – ein besonderes Problem bei jüngeren Patienten mit langer Lebenserwartung [12, 13].

In den letzten 15 Jahren hat sich der subkutane ICD (S-ICD) als echte Alternative etabliert. Er vermeidet intravasale Elektroden, bietet einen zuverlässigen Schutz vor ventrikulären Arrhythmien und reduziert das Risiko für systemische Infektionen und die genannten Langzeitkomplikationen erheblich. Parallel schreitet die Risikostratifizierung weiter voran: Moderne Bildgebung, genetische Tests, klinische Scores und zunehmend auch KI(künstliche Intelligenz)-basierte Ansätze helfen, jene Patienten zu identifizieren, die am meisten von einer Defibrillatortherapie profitieren.

Diese Übersichtsarbeit fasst den aktuellen Wissensstand zum S‑ICD zusammen, diskutiert Implantationstechniken, relevante Studiendaten und die Bedeutung einer sorgfältigen Patientenselektion für die klinische Praxis.

## Das S-ICD-System – Historie und Status quo

**Abb. 1:**
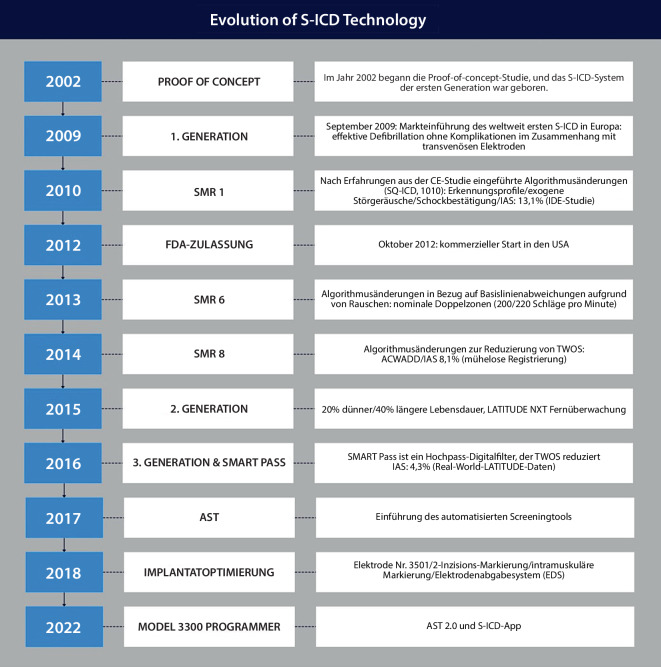
Evolution des subkutanen implantierbaren Kardioverter-Defibrillators (*S‑ICD*). *FDA* Food and Drug Administration, *SMR* „software maintenance release“, *AST* automatisiertes Screeningtool, *ACWADD* „alternating correlation waveform appraisal – double detection“, *TWOS* „T-wave oversensing“, *IAS* inadäquater Schock

Die Wirksamkeit des subkutanen Defibrillators unter der Rationale einer Vermeidung intrakardialer Elektroden wurde ab 2002 in ersten Proof-of-Concept-Untersuchungen bestätigt mit der ersten Gerätegeneration SQ^TM^-ICD 1010 (Cameron Health Inc., San Clemente, CA, USA [14]). Der subkutane ICD (S-ICD®) wurde 2008 erstmals dauerhaft implantiert. Das System umfasst einen links-lateral intermuskulär platzierten Impulsgenerator und eine episternal platzierte subkutane Elektrode mit 8‑cm-Schockcoil. Frühe Studien zeigten mittlere Defibrillationsschwellen um 35 J; zur Sicherheitsmarge wurde der Output auf 80 J festgelegt – mit deutlich größerem Aggregat im Vergleich zum transvenösen (TV-) ICD. Das FDA(Food and Drug Administration)-Approval erfolgte 2012. In der eigenen Klinik (Universitätsmedizin Mannheim, UMM) wurde das Konzept der nichttransvenösen Defibrillation bereits ab den frühen 2000er-Jahren wissenschaftlich und klinisch untersucht mit einem konkurrierenden System (Medtronic; Solo-Chili-Projekt), mit dem durch eine dorsale subkutane Fingerelektrode und einem kaudal gelagerten subpektoralen Defibrillator eine Schockeffektivität von 96 % erreicht wurde [15]. Die ersten S‑ICD in der UMM wurden 2010 implantiert und im Verlauf ein S‑ICD-First-Ansatz etabliert sowie ein internationales Ausbildungs- und Proktorenzentrum für diese Therapie gegründet mit mittlerweile mehr als 1000 implantierten S‑ICD. Über die Jahre erfolgten dann zahlreiche technische und prozedurale Weiterentwicklungen. Als relevante Eckdaten seien genannt (Abb. 1):

- 2. Generation (EMBLEM S‑ICD; ab 2015): um ca. 20 % kleineres Aggregat, um ca. 40 % längere Batterielaufzeit; Einführung von Remote-Monitoring.
- 3. Generation (EMBLEM MRI S‑ICD; ab 2016): MRT(Magnetresonanztomographie)-Kompatibilität/MRI („magnetic resonance imaging“) Protection Mode, Vorhofflimmer-Monitoring, SMART Pass (zuvor ab 2014 bereits ACWADD [„alternating correlation waveform appraisal – double detection“]) zur Reduktion inadäquater Therapien durch T‑Wellen-Oversensing (TWOS). Eine 4. Generation mit neuer Plattform ist in Entwicklung und wird ca. 2029 erwartet.
- Implantationstechnik: Übergang von der ursprünglichen Drei- zur Zweischnitttechnik (seit 2013) mit kürzerer Prozedurzeit, günstigerem Infektions‑/Kosmetikprofil und breiter Adoption.
- Aggregat‑/Taschenlage: Intermuskuläre bzw. in Einzelfällen submuskuläre Platzierung verbessert Effizienz, reduziert inadäquate Wahrnehmungen und Auslösungen und verringert Taschenkomplikationen, v. a. bei sehr schlanken oder adipösen Patienten.
- Signalverarbeitung und Programmierung: Zwei-Zonen-Programmierung („conditional and shock zone“) und der Hochpassfilter SMART Pass (Boston Scientific) verringern TWOS und inadäquate Schocks (IAS); aktuelle Studien berichten ca. 3–4 % IAS nach 1 Jahr [16].

Das aktuelle System besteht aus dem Impulsgenerator (IPG; EMBLEM S‑ICD, Boston Scientific) und der subkutanen Elektrode 3501. Der Impulsgenerator dient sowohl der Arrhythmiedetektion als auch der Abgabe der Hochvolttherapie. Das Titangehäuse ist als aktive Schockelektrode ausgelegt und liefert eine maximale Schockenergie von 80 J. Eine kontinuierliche Bradykardiestimulation oder antitachykardes Pacing (ATP) sind nicht möglich, jedoch steht eine optionale Post-Schock-Bradykardiestimulation (50 ppm, bis 30 s) zur Verfügung. Die durchschnittliche Batterielaufzeit beträgt 8 bis 9 Jahre.

Die Elektrode wird subkutan parasternal getunnelt und parallel zum Sternum in Fasziennähe positioniert, um einen optimalen Defibrillationsvektor zwischen Elektrode und Aggregat zu erzeugen. Sie verfügt über 2 Detektionspole, aus denen – gemeinsam mit dem Aggregat – 3 Detektionsvektoren (primär, sekundär, alternativ) gebildet werden. Die 8 cm lange Defibrillations-Coil verläuft über dem Sternum.

**Abb. 2:**
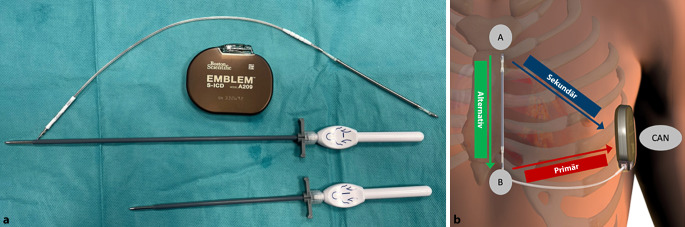
**a** Impulsgenerator (IPG) EMBLEM A209, Elektrode Modell 3501 und EMBLEM-S-ICD(subkutaner implantierbarer Kardioverter-Defibrillator)-Elektroden-Einführsystem (EDS), **b** Lage des S‑ICD und der Elektrode sowie verfügbare Vektoren (primär, sekundär, alternativ)

Die wichtigsten technischen Spezifikationen sind in Tab. 1 zusammengefasst. Eine Übersicht der Systemkomponenten zeigt Abb. 2.

**Tab. 1:** Technische Spezifikationen des EMBLEM-S-ICD(subkutaner implantierbarer Kardioverter-Defibrillator)-Systems

Komponente	Spezifikation
Impulsgenerator (A209/219) 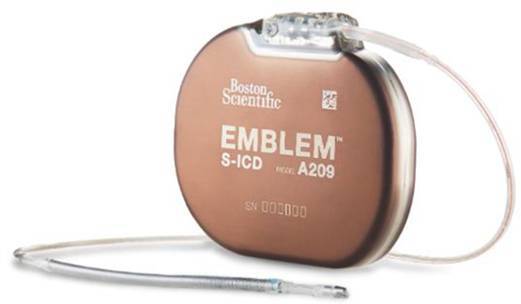	Maße: 83,1 × 69,1 × 12,7 mm
	Volumen: 59,5 cm^3^
	Gewicht: 130 g
	Material: Titan mit Titannitritbeschichtung (aktive Schockelektrode)
	Abgegebene Energie: bis 80 J
	Stimulation: keine Dauerstimulation/ATP; Post-Schock-Bradykardiestimulation: 50 ppm bis 30 s
	Batterielaufzeit: ca. 8–9 Jahre
Elektrode 3501	Länge: 45 cm
	Material: Polycarbonat/Polyurethanisolierung
	Defibrillationsoberfläche: 750 mm^2^
	Schäfte: 7 F (Schaft), 9 F (Coil), 11,5 F (distale Spitze)
	Detektion: 2 Pole (distal/proximal), 3 Detektionsvektoren
	Implantation: subkutan, parasternal (1–2 cm links der Mittellinie)
	Verbindung: SQ-1-Stecker

*SQ* Subcutaneous, *ATP* antitachykardes Pacing

Die Geräteabfrage und -programmierung erfolgt über das LATITUDE™-Programmiersystem Modell 3300. Das EMBLEM™-S-ICD™-System ist mit LATITUDE™ NXT kompatibel; Remote-Monitoring überträgt u. a. Batteriestatus, Systemimpedanz sowie Episoden‑/SMART-Pass-Informationen. Der S‑ICD® ist MRI-kompatibel bei 1,5 T.

## Datenlage und Evidenz zum S-ICD

Die Evidenz zum S‑ICD ist inzwischen eindrucksvoll: In randomisierten Vergleichsstudien, großen prospektiven Kohorten und Registern sind zusammen mehr als 5000 Patientinnen und Patienten erfasst (u. a. PRAETORIAN *n* = 849; UNTOUCHED *n* = 1111; EFFORTLESS *n* = 984; PAS *n* = 1637, ATLAS *n* = 503; u. v. m.). Über die Studien hinweg ergeben sich eine hohe Schockeffektivität um etwa 98 %, niedrige Raten an IAS unter zeitgemäßer Programmierung und – im Vergleich zum TV-ICD – ein deutlich günstigeres Komplikationsprofil, insbesondere bei elektrodenassoziierten Ereignissen. PRAETORIAN wies für den kombinierten Endpunkt aus gerätebezogenen Komplikationen und IAS die Nichtunterlegenheit des S‑ICD nach [17]; Sekundär- und Langzeitdaten zeigen weniger Major- und v. a. weniger elektrodenassoziierte Komplikationen unter S‑ICD [18]. In PRAETORIAN-XL (bis 8 Jahre) waren in der As-treated-Analyse sämtliche Komplikationen seltener [19]. ATLAS ergänzte dies um eine Überlegenheit bei perioperativen, elektrodenbedingten Komplikationen in einer jüngeren Patientenpopulation mit überwiegend hereditären Kardiomyopathien/Kanalerkrankungen bei gleichzeitig erhaltener Schockeffektivität [20]. UNTOUCHED bestätigte im primärpräventiven Hochrisikokollektiv eine sehr hohe Schockeffektivität und niedrige IAS-Raten bei standardisierter Programmierung mit hohen Cut-offs der VT-Zonen [16]. EFFORTLESS lieferte 5‑Jahres-Daten mit hoher Schockeffektivität spontaner Episoden und Sicherheit der Elektroden [21]; die große FDA-Post-Approval-Studie (PAS) zeigte unter Real-World-Bedingungen eine Systemkomplikationsfreiheit von mehr als 93 % und eine Elektrodenkomplikationsfreiheit von etwa 99 % [22]. In der Summe sind IAS mit moderner Programmierung („dual zone“, SMART Pass) auf etwa 3–5 % nach 1 Jahr gesunken; die Ursachen unterscheiden sich dabei allerdings systemtypisch (S-ICD häufiger T‑Wellen‑/Low-Amplitude-Oversensing, TV-ICD häufiger supraventrikulär getriggerte IAS; [23]). Aktuell wurde als Late-Breaker auf dem ESC(European Society of Cardiology)-Kongress eine individuelle Patientendatenmetaanalyse von PRAETORIAN und ATLAS (*n* = 1342) vorgestellt, die bei insgesamt niedrigen Raten an IAS ein höheres Risiko für den ersten IAS unter S‑ICD versus TV-ICD (2,5 vs. 1,5 %/Jahr; Hazard Ratio [HR]: 1,61; 95 %-Konfidenzintervall [KI]: 1,06–2,45) zeigte, getriggert durch kardiales Oversensing und elektromagnetische Interferenzen (EMI), während atrial tachyarrhythmisch verursachte IAS seltener waren; die Gesamtlast inadäquater ICD-Therapie (ATP/Schock) unterschied sich nicht signifikant. Eine weitere PRAETORIAN-Auswertung zeigte eine vergleichbare Lebensqualität im Vergleich von S‑ICD und TV-ICD; Schocks beeinträchtigen die Lebensqualität unabhängig vom implantierten System (TV-ICD und S‑ICD), somit bleibt die Schockreduktion (Programmierung/Filter, Patientenedukation zu EMI) zentral [24].

Weiterhin sind Art und Schwere von Komplikationen unterschiedlich: Unter S‑ICD treten klassische Elektrodenprobleme (> 90 %ige Reduktion) und systemische Infektionen (0 %) deutlich seltener auf; S‑ICD-Komplikationen erfordern seltener invasive Eingriffe oder Hospitalisationen [17, 19]. Insgesamt ist die Wirksamkeit des S‑ICD mindestens gleichwertig, die Sicherheit – insbesondere elektrodenassoziiert – günstiger.

Eine auszugsweise Übersicht wichtiger Studien/Register ist Tab. 2 zu entnehmen.

**Tab. 2:** Evidence Snapshot: S‑ICD®-Kernstudien und Registerdaten auf einen Blick

Studie (Jahr/*n*)	Design und Follow-up	Primärer Endpunkt	Zentrale Ergebnisse (ausgewählte Kennzahlen)
PRAETORIAN [17] (2020)/*n* = 849	Randomisiert S‑ICD vs. TV-ICD; medianes FU 49 Monate. Nichtunterlegenheit	Komposit: gerätebedingte Komplikationen und IAS	Nichtunterlegenheit erreicht (HR: 0,99; 95 %-KI: 0,71–1,39). Komplikationen: 5,9 % (S-ICD) vs. 9,8 % (TV-ICD); IAS: 9,7 % vs. 7,3 %
PRAETORIAN-XL [19] (2025)/*n* = 849	Langzeit-FU bis 8 Jahre (verlängerte Kohorte)	Komplikationen (Langzeitbetrachtung)	8‑Jahres-mITT: keine Signifikanz für alle Device-Komplikationen (8,0 % vs. 11,6 %; *p* = 0,15), aber TV-ICD mit signifikant mehr Major- (*p* = 0,03) und elekrodenassoziierten Komplikationen (*p* < 0,001). „As treated“: Vorteil S‑ICD (HR: 0,64; *p* = 0,047)
UNTOUCHED [16] (2021)/*n* = 1111	Prospektiv, Primärprävention (LVEF ≤ 35 %), standardisierte Programmierung; FU 18 Monate	IAS-Freiheit nach 18 Monaten (vs. 91,6 % Performance-Ziel)	IAS sehr niedrig: 1‑Jahres-Gesamt: 3,1 %; mit Gen-3/SMART Pass 2,4 %. 18 Monate IAS-frei: 95,9 % (Performance-Ziel erreicht). Finale Schockkonversion: 98,4 %; Komplikationsfreiheit: 92,7 %
EFFORTLESS [21] (Langzeitregister)/*n* ≈ 984	Multizentrisches Register; 1. Generation S-ICD; Langzeitdaten > 5 Jahre publiziert	Sicherheit und Wirksamkeit im Versorgungsalltag	Frühe 1‑Jahres-IAS: 8,1 %. Nach 5 Jahren anhaltend hohe Wirksamkeit (ca. 98 % Terminierung spontaner VT/VF) bei günstigem Sicherheitsprofil. Neuere Programmierung senkt IAS weiter
PAS (JACC 2023) [22]/*n* = 1637	FDA-Post-Approval-Studie, prospektiv, 5‑Jahres-FU	Sicherheits‑/Wirksamkeitsziele (Komplikationsfreiheit, Schockeffektivität)	Systemkomplikationsfreiheit: 93,4 %; Elektrodenkomplikationsfreiheit: 99,3 %; Initial‑/Final-Schockeffektivität: 98,4 %. IAS kumulativ: 15,8 % in 5 Jahren (ca. 6,7 % im 1. Jahr). Upgrade zu TV-ICD wegen Pacing: 1,6 %
ATLAS (2022) [20]/*n* ≈ 503	Randomisiert S‑ICD vs. TV-ICD (jüngere Patienten, hereditäre Kardiomyopathien/Kanalerkrankungen)	Perioperative, elekrodenassoziierte und Major-Komplikationen (bis 6 Monate)	Deutlich reduziert mit S‑ICD: 0,4 % vs. 4,8 % (*p* = 0,001). Trend zu mehr IAS mit S‑ICD
Sekundäranalysen [18, 23, 24, 45] (2022–2024)	PRAETORIAN-Subanalysen, *European Heart Journal* 2022 Circulation EP 2024 Circulation Cardiovascular Quality and Outcome 2024	Komplikationsprofil, IAS-Muster, Lebensqualität	TV-ICD: mehr invasive/elektrodenbedingte Komplikationen; S‑ICD: IAS eher Oversensing/EMI, TV-ICD: häufiger SVT/ATP-getrieben (inkl. inadäquater ATP). QoL insgesamt ähnlich

*S‑ICD* subkutaner implantierbarer Kardioverter-Defibrillator, *EMI* elektromagnetische Interferenzen, *TV-ICD* transvenöser ICD, *IAS* inadäquater Schock, *AS* adäquater Schock, *ATP* antitachykardes Pacing, *FU* Follow-up, *EMI* elektromagnetische Interferenz, *VF* „ventricular flutter“, *VT* ventrikuläre Tachykardie, *SVT* supraventrikuläre Tachykardie, *QoL* „quality of life“ (Lebensqualität), *mITT* „(modified) intention to treat“, *HR* Hazard Ratio, *(s)HR* „(subdistribution) HR“, *KI* Konfidenzintervall, *LVEF* linksventrikuläre Ejektionsfraktion

## Patientenselektion und Implantation des subkutanen Defibrillators

### Patientenselektion

Ausgangspunkt ist die gesicherte ICD-Indikation. Liegt kein Bedarf an Bradykardiestimulation, antitachykarder Stimulation (ATP) oder kardialer Resynchronisation (kardiale Resynchronisationstherapie, CRT) vor, ist der S‑ICD grundsätzlich für alle Patienten mit einer ICD-Indikation eine regelhafte Option. Die verfügbare Evidenz (randomisierte Studien, Register) stützt diesen Ansatz. Upgrade-Raten wegen späteren Pacing-Bedarfs sind insgesamt niedrig; so lag der Crossover in den transvenösen Arm in der PRAETORIAN-Studie (Follow-up: 49 Monate) nur bei 1,2 % für Pacing- und bei 1,4 % für CRT-Bedarf [17, 19]. Ein gerne angeführtes Argument gegen den S‑ICD ist, dass der Betroffene mit Herzinsuffizienz noch nicht maximal mit Betablockern aufdosiert ist und im Verlauf stimulationsabhängig werden könnte, was manche Anwender sogar dazu verleiten lässt, präventiv transvenöse Zweikammersysteme zu implantieren. Dies ist seit 2016 in den ESC Heart Failure Guidelines explizit kontraindiziert (Klasse III), hier steht vielmehr die Anpassung der Betablockerdosis im Vordergrund [7].

Darüber hinaus ist der S‑ICD naturgemäß besonders naheliegend auch bei jüngeren Patienten mit langer Lebenserwartung und aktivem Lebensstil, bei denen das kumulative Risiko elektrodenassoziierter Ereignisse besonders ins Gewicht fällt [20]. Er empfiehlt sich ebenso bei erhöhtem Infektionsrisiko oder vorausgegangener Systeminfektion, unter Dialyse, bei Immunsuppression oder bei vorhandenen Kunstklappen sowie bei erschwertem venösen Zugang oder komplexer Anatomie einschließlich angeborener Herzfehler. Eine weitere Kernzielgruppe bilden Patienten mit hereditären Arrhythmiesyndromen und Kardiomyopathien ohne absehbaren Pacing-Bedarf (z. B. hypertrophe [HCM] und arrhythmogene rechtsventrikuläre Kardiomyopathie [ARVC], Brugada- und Long-QT-Syndrom; [25]). Nicht zuletzt ist der S‑ICD eine Option für Patienten, die im Rahmen des Shared Decision Making ausdrücklich eine extravaskuläre Lösung bevorzugen.

### Präoperatives Screening

**Abb. 3:**
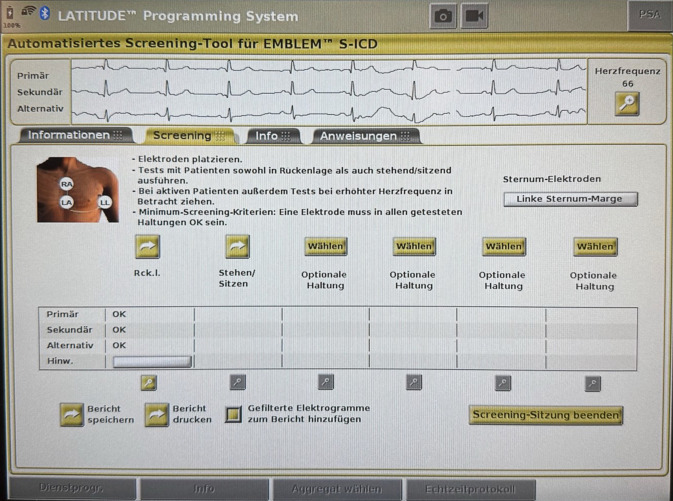
Automatisiertes Screeningtool (*AST*) zur präoperativen Überprüfung der Eignung des Patienten für den subkutanen implantierbaren Kardioverter-Defibrillator (*S‑ICD*): In diesem Fall sind alle 3 Vektoren geeignet

Wegen der Fernfeldsignalverarbeitung (ähnlich dem Oberflächen-EKG) ist ein standardisiertes EKG-Screening mit dem mittlerweile im Programmer integrierten automatisierten Screeningtool (AST) empfohlen (Abb. 3). Gefordert ist mindestens 1 stabiler Sensing-Vektor in verschiedenen Körperpositionen. Bei Patienten ohne Pacing-Indikation werden in der Regel hohe Eignungsraten von mehr als 90–95 % erreicht. Faktoren für ein negatives Screening sind u. a. ausgeprägte T‑Wellen-Morphologien (z. B. bei HCM) oder ein ungünstiges R/T-Verhältnis. Rechtsparasternal platzierte Elektroden können die Screeningergebnisse bei HCM und Brugada-Syndrom verbessern. Da HCM und ARVC progredient sein können, ist eine erneute Bewertung im Verlauf sinnvoll. Demgegenüber sind unter transvenösen Systemen bei ARVC gehäuft elektrodenassoziierte Probleme beschrieben, was ein klares Argument für den S‑ICD ist [26].

Vorschlag zum praktischen Entscheidungsweg:

- ICD-Indikation klären (Primär‑/Sekundärprävention); bei nichtischämischer Kardiomyopathie (NICM) ergänzend Bildgebung/Genetik/Klinik einbeziehen – unabhängig vom Systemtyp.
- Stimulationsbedarf prüfen: Wenn Pacing/ATP/CRT absehbar, transvenöses System, wenn nicht, S‑ICD abwägen/bevorzugen.
- Risiko-Nutzen abwägen: Infektionsrisiko, venöse Anatomie, Lebenserwartung, berufliche/athletische Belastungen und Patientenpräferenzen.
- Screening durchführen und, falls geeignet, Implantation planen; Programmierung mit hohen Therapie-Cut-offs und 2‑Zonen-Strategie.
- Nachsorge: Idealerweise telemedizinische Einbindung und gezielte Nachprogrammierung (inkl. verfügbarer Filter) zur Minimierung inadäquater Therapien.

### Implantation des S-ICD

Die Implantation des S‑ICD nach Best-Practice-Vorgaben ist entscheidend und erfolgt orientiert an anatomischen Strukturen ohne Notwendigkeit einer intraoperativen Fluoroskopie. Eine präoperative Fluoroskopie zur Optimierung von (eingezeichneter und aufgelegter) Generator- und Elektrodenlage ist empfohlen. Historisch wurde das System in einer Dreischnitttechnik implantiert (links-lateral für die Aggregattasche sowie inferior-und superior-parasternal für die Elektrode). Seit 2013 hat sich die 2‑Inzisions-Technik etabliert: Die obere parasternale Inzision entfällt, die Elektrode wird von der unteren xyphoidalen Inzision aus über dem Sternum mit einem 11-F-Peel-Away-Sheath getunnelt. Dieser Ansatz verkürzt die Prozedur, senkt das Infektionsrisiko und verbessert die Kosmetik und Komplikationsraten [27].

**Abb. 4:**
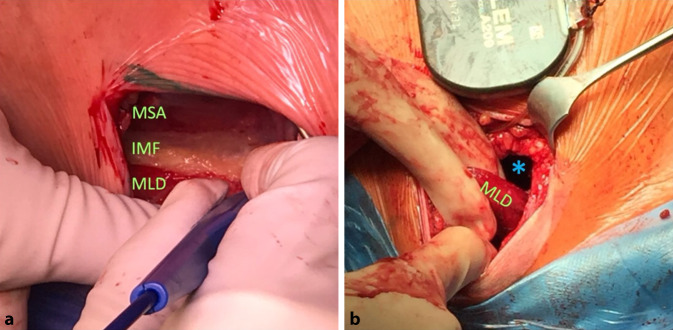
Intermuskuläre Tasche: **a** intermuskuläre Faszie (*IMF*) zwischen M. serratus anterior (*MSA*) und M. latissimus dorsi (*MLD*), **b** freigelegter M. latissimus dorsi und intermuskuläre Tasche (*Asterisk*) für die Aggregatpositionierung

Bezüglich der Aggregatlage ist die Bezeichnung „subkutaner“ Defibrillator irreführend. Klar favorisiert wird eine intermuskuläre Lage zwischen M. serratus anterior und latissimus dorsi (Abb. 4) mit nachgewiesen verbesserter Schockgeometrie, reduzierten Taschenkomplikationen wie Erosion oder Schmerzen sowie einem günstigen kosmetischen Ergebnis auch bei sehr schlanken Patienten und vermindertem Migrationsrisiko [28]. Auch eine submuskuläre Implantation (unter dem M. serratus anterior) ist möglich, aber technisch anspruchsvoller und erfordert besondere Sorgfalt hinsichtlich des N. thoracicus longus sowie ein adäquates Analgesiekonzept [29]. Eine Kurzdarstellung der Taschenpräparation und der Tunnelierungen ist den Online-Videos 1 bis 3 zu entnehmen.

**Abb. 5:**
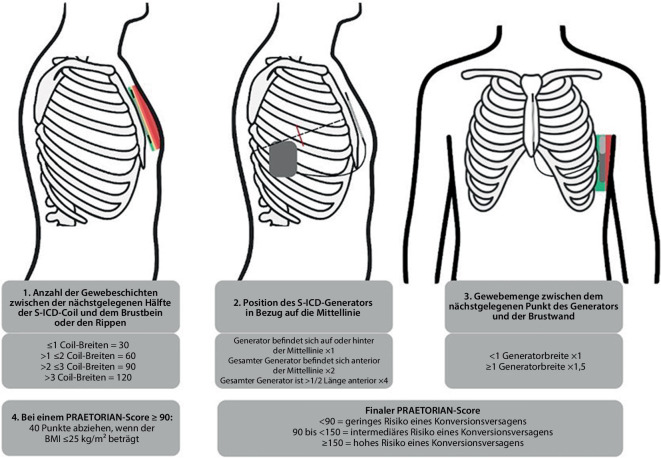
PRAETORIAN-Score zur Evaluierung einer guten Aggregat- und Elektrodenlage sowie zur Prädiktion der Schockeffektivität: Ein Score < 90 hat eine Wahrscheinlichkeit von ca. 99 % für eine erfolgreiche Defibrillation. *S‑ICD* subkutaner implantierbarer Kardioverter-Defibrillator, *BMI* Body-Mass-Index. (Mod. nach [30])

**Abb. 6:**
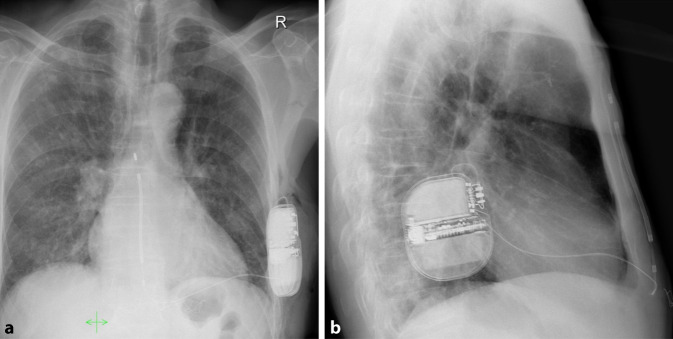
Postoperatives Röntgenbild in 2 Ebenen (**a** posterior-anterior, **b** lateral) mit idealer Systemlage und einem PRAETORIAN-Score von 30, Systemimpedanz 58 Ω

Die Defibrillationsschwellentestung (DFT) wird aktuell noch empfohlen, da suboptimale Implantpositionen die Schwelle erhöhen und das Konversionsrisiko verschlechtern können. Prädiktoren einer erhöhten Schwelle sind insbesondere der Abstand der Schock-Coil zum Sternum, die Gewebeschicht zwischen Aggregat und Thoraxwand, die anteroposteriore Aggregatposition sowie der Body-Mass-Index. Diese Determinanten fließen in den PRAETORIAN-Score ein (Abb. 5), der anhand postoperativer Thoraxaufnahmen berechnet wird und Patienten mit suboptimaler Lage identifiziert [30]. Ein PRAETORIAN-Score von weniger als 90 weist in der laufenden PRAETORIAN-DFT-Studie einen negativen prädiktiven Wert („negative predictive value“, NPV) von 99 % für ein DFT-Versagen auf (also ca. 99 % Erfolgswahrscheinlichkeit der DFT; [31]). Der „gefühlte“ PRAETORIAN-Score intraoperativ (Gerät liegt intermuskulär, dorsale Aggregatlage und eindeutiges Tunnelieren auf dem Sternum) korreliert dabei sehr gut mit dem postoperativen Röntgen [32]. Abb. 6 zeigt ein postoperatives Röntgenbild in 2 Ebenen mit idealer Systemlage und einem PRAETORIAN-Score von 30.

Nach dem ohmschen Gesetz beeinflusst die Systemimpedanz („high voltage“/„low-voltage“) die DFT entscheidend: Bei gegebener Schockenergie führt eine niedrigere Impedanz (ideal < 90 Ω) zu höherem Spitzenstrom und größerer elektrischer Feldstärke zwischen Elektrode und Aggregat, was die Konversion begünstigt. Isolierte Impedanzwerte sind allerdings kein ausreichender Prädiktor, sie sollten stets zusammen mit der Implantatposition (PRAETORIAN-Score) beurteilt werden. Eine zu anteriore Aggregatlage kann trotz niedriger Impedanz zu ineffektiver Defibrillation führen, da die Energie über den rechten Ventrikel abdrainiert wird.

Im Hinblick auf die Anästhesie ist, verglichen mit dem TV-ICD, aufgrund der größeren Dissektion für die Tasche und die Tunnelierungen sowie der DFT eine tiefere Analgesie/Sedierung erforderlich. Während nur noch wenige Zentren in Allgemeinanästhesie implantieren, sind MAC(„monitored anesthesia care“)-Verfahren (tiefe Analgosedierung) und seltener (zusätzlich) regionale Blockaden (z. B. Serratus anterior/Transversus thoracis) heutzutage führend [33].

Aggregatwechsel sind ebenfalls unkompliziert unter Eröffnung der alten Tasche durchzuführen. Regelhaft beobachtete Anstiege der Impedanz über die Zeit (durch Kapselbildung etc.) sind ohne klinische Relevanz für die Schockeffektivität. Kapselektomien können die Impedanz senken, sind aber mit höheren Blutungsrisken verbunden [34, 35].

Eine genaue Anleitung zur Implantation, welche auf eine frühe Initiative des Academic Medical Center (AMC) Amsterdam und der Universitätsmedizin Mannheim mit Boston Scientific entstand, ist unter der Educare-Homepage von Boston Scientifc nach vorheriger Registrierung abrufbar (https://educare.bostonscientific.eu).

## Modulares Konzept mit kommunizierenden „leadless pacemakers“

**Abb. 7:**
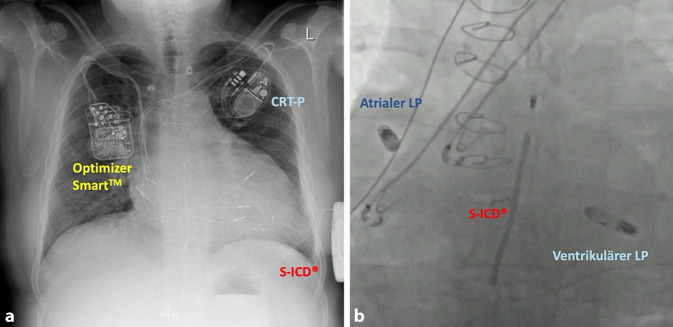
Kombination des subkutanen implantierbaren Kardioverter-Defibrillators (*S‑ICD*) mit anderen kardialen implantierbaren Devices (jeweils adäquate Gerätefunktion ohne Interferenzen oder Crosstalk): **a** mit einem Gerät zur kardialen Kontraktilitätsmodulation (Optimizer Smart^TM^, Impulse Dynamics) und einem biventrikulären Schrittmacher (*CRT‑P*), **b** mit einem Dual Chamber Leadless Pacemaker (*LP*; AVEIR^TM^ DR, Abbott)

Die zentrale Limitation des S‑ICD besteht in der fehlenden Stimulationsfunktion. Weder ATP noch eine kontinuierliche Bradykardiestimulation sind integriert (abgesehen von einer kurzzeitigen Post-Schock-Stimulation). Eine Kombination mit anderen kardialen implantierbaren Systemen („cardiac implantable eletronic devices“, CIED) ist unter bestimmten Voraussetzungen möglich (Abb. 7) und wurde von der eigenen Arbeitsgruppe im Detail beschrieben [36]. Speziell sollte ein Crosstalk-Test mit maximalem Stimulationsoutput durchgeführt und ein manuelles Template unter aktiver Stimulation hinterlegt werden. Bei Patienten mit transvenösen Schrittmachersystemen ist zudem die unipolare Sicherheitsumschaltung zu deaktivieren, um eine dauerhafte bipolare Stimulation sicherzustellen. Andernfalls könnte eine unipolare Stimulation zur Inhibition der Schockabgabe des S‑ICD führen [37].

**Abb. 8:**
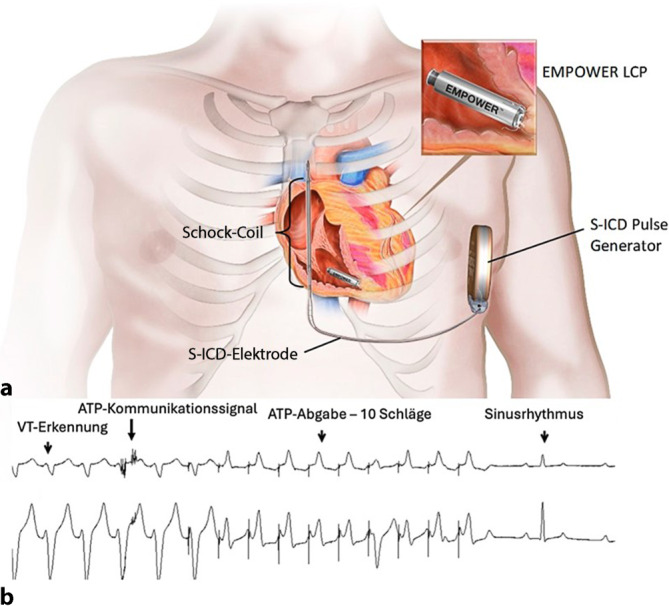
Kommunizierendes modulares S‑ICD(subkutaner implantierbarer Kardioverter-Defibrillator)-LP(„leadless pacemaker“ [EMPOWER])-System: **a** Kombination des S‑ICD mit dem EMPOWER (jeweils Boston Scientific), **b** erfolgreiche ATP(antitachykarde Stimulation)-Abgabe bei einer induzierten ventrikulären Tachykardie (VT). (Mit freundlicher Genehmigung von Boston Scientific, mod. nach [41])

Durch Kombination des S‑ICD mit einem kabellosen VVI(Stimulationsort: Ventrikel, Sensing-Ort: Ventrikel, Betriebsmodus: Inhibition)-Schrittmacher (EMPOWER^TM^ LP [„leadless pacemaker“], Boston Scientifc) wird diese Lücke zukünftig im Sinne eines kommunizierenden mCRM(„modular cardiac rhythm management“)-Systems geschlossen. Bei klinischem Bedarf kann der EMPOWER^TM^ zusätzlich implantiert werden, der ATP oder eine antibradykarde Stimulation abgeben kann (Abb. 8). Die drahtlose Kommunikation der Devices erfolgt über das Feld zwischen Schock-Coil und Aggregat, eine apikoseptale RV-Position des LP begünstigt dabei die Signalaufnahme [38]. Präklinisch wurden Kommunikationsstabilität, Pacing-Performance und Explantations‑/End-of-life-Strategien umfassend untersucht [39–42].

Klinisch liegt mit MODULAR-ATP [43] eine globale, multizentrische Studie vor (Single-Arm, bis 300 Patienten). In die bisherige Analyse wurden 293 Patienten eingeschlossen; bei allen konnte ein mCRM-System erfolgreich implantiert werden (41 % LP-Add-on zu bestehendem S‑ICD, 59 % Kombiimplantation). Im Median dauerten die Eingriffe etwa 35 min (LP) bzw. ungefähr 79 min (Kombi); eine intraprozedurale LP-Reposition war in etwa 27 % der Fälle nötig. Eine DFT erfolgte bei zirka 94 %, eine Erstschockkonversion bei etwa 94 %. Entscheidend war, dass die unidirektionale Kommunikation zwischen S‑ICD und LP in 98,8 % aller Tests funktionierte; Reizschwelle und R‑Wellen blieben jeweils stabil. ATP wurde bei 31 Episoden von 13 Patienten abgegeben, die Terminierungsrate lag bei 61 %. Nicht beendete Episoden terminieren spontan oder wurden geschockt; Kommunikationsausfälle als Therapieversagen traten nicht auf. IAS (ca. 5 %) betrafen überwiegend Oversensing langsamer VT. Für die Nachbeobachtung von 6 Monaten wurde eine Freiheit von LP-bezogenen Major-Komplikationen von 97,5 % bestätigt [44].

Wie groß ist aber nun der klinische ATP-Bedarf unter zeitgemäßer ICD-Programmierung wirklich? Subanalysen aus PRAETORIAN zeigen, dass S‑ICD-Patienten – mangels ATP – zwar häufiger einen adäquaten Schock erhalten, die Gesamtzahlen adäquater Schocks sich jedoch nicht unterscheiden [45]; ATP kann im Gegenzug Arrhythmien beschleunigen und Storm-Episoden begünstigen. Parallel legt die APPRAISE-ATP-Studie in einer primärpräventiven TV-ICD-Population eine relative Reduktion der Zeit bis zum ersten Schock unter „ATP-on“ nahe (ca. 28 %, *p* = 0,005), bei allerdings nur sehr kleinem absoluten Zusatznutzen (ca. 1 %/Jahr) ohne Unterschied in der Gesamtschockzahl; das Risiko für VT/VF-Storm war unter „ATP-on“ mehr als verdoppelt [46]. Die ENHANCED-ICD-Studie belegt, dass eine programmierte Detektionsverzögerung sicher ist und unnötige Therapien vermeidet (langfristig um ca. 17 %). Zusammen mit MADIT-RIT/PREPARE/ADVANCE III [47–49] stützt dies einen defensiven, verzögerten Therapieansatz, wie auch in den Leitlinien empfohlen [50].

Einordnung für die Praxis: Modular ATP/EMPOWER schließt die funktionale Lücke des S‑ICD „on demand“ und erhält trotzdem dessen strukturellen extravaskulären Vorteil. Patienten ohne absehbaren Pacing‑/ATP-Bedarf erhalten zunächst den S‑ICD, bei Auftreten ATP-sensitiver VT oder von Bradykardien wird der LP ergänzt. Vor dem Hintergrund moderner Programmierung und der APPRAISE-ATP-Ergebnisse spricht dabei viel für eine selektive, nicht generelle ATP-Strategie; das modulare mCRM-Konzept bildet diese Logik passgenau ab. Bezüglich der Bradystimulation muss einschränkend gesagt werden, dass es sich naturgemäß um eine asynchrone rechtsventrikuläre Einkammerstimulation handelt. Idealerweise sollte dies perspektivisch auch um physiologischere Stimulationsformen ergänzt werden (CSP[„conduction system pacing“]/CRT). Die CE(Conformité Européenne)-Zulassung wird etwa 2027 erwartet.

## Zukunft der S-ICD-Therapie

Der S‑ICD hat im Vergleich zum TV-ICD seine Gleichwertigkeit in der Terminierung ventrikulärer Tachyarrhythmien bewiesen und bietet zugleich ein günstigeres Sicherheitsprofil, insbesondere bei elektrodenassoziierten und interventionspflichtigen Komplikationen [18, 19]. Die aktuellen Leitlinien (American College of Cardiology [ACC]/American Heart Association [AHA]/Heart Rhythm Society [HRS] 2017; ESC 2022) empfehlen ihn bei fehlendem Stimulationsbedarf (Brady-Pacing/ATP/CRT; [6, 51]). Mit zeitgemäßer Programmierung sowie neuen Algorithmen und Filtern (SMART Pass) sind IAS in den letzten Jahren weiter zurückgegangen [16, 23]. Vor diesem Hintergrund ist mit einer Neubewertung und sehr klaren Positionierung des S‑ICD in künftigen Leitlinien zu rechnen, insbesondere da die Langzeitanalysen der PRAETORIAN-Studie auf einen Überlegenheitsnachweis ausgelegt sind.

Randomisierte und prospektive Daten zeigen, dass ein generelles „ATP-für-alle“ keinen Zusatznutzen bei der Gesamtschocklast bringt – zumal ATP Arrhythmien beschleunigen kann [46]. Genau hier setzen zukünftig modulare Konzepte an, bei denen ein LP (EMPOWER) bei Bedarf ergänzt wird. Die frühe klinische Erfahrung ist technisch überzeugend; längere Follow-ups werden die Nachhaltigkeit klären [44].

Mit der nächsten Gerätegeneration in einigen Jahren sind auch modifizierte Gerätedesigns (Form, Größe) vorstellbar. DFT-Untersuchungen, abhängig vom PRAETORIAN-Score, zeigen, dass die mittlere Defibrillationsschwelle bei optimaler Lage deutlich unter dem aktuell verfügbaren 80-J-Output liegt [52]. Experimentelle Ansätze mit zusätzlichen Schock-Coils senken die DFT weiter signifikant um mehr als 30 %, sind aber (noch) kein Alltag [53]. Parallel adressiert die laufende PRAETORIAN-DFT-Studie die Frage, wann eine DFT bei optimaler Position sicher entfallen kann, was Logistik und Narkosebedarf spürbar vereinfachen würde [31, 54]. In der Praxis wird bereits jetzt häufig auf eine DFT ohne negativen Einfluss auf das Outcome verzichtet [55, 56]. Potenzial zur weiteren Reduktion von IAS liegt in der perspektivisch potenziellen Diskriminierung über alle 3 verfügbaren Vektoren (und ggf. über Signale des EMPOWER).

Schließlich bleibt der S‑ICD strategisch flexibel: Er bewahrt den venösen Zugang für die Zukunft. Entsteht später ein Pacing- oder CRT-Bedarf, ist das Crossover auf ein transvenöses System möglich, insbesondere da das S‑ICD-System einfach und komplikationsarm zu explantieren bzw. zu extrahieren ist (Online-Video 4).

Als wichtigste Hürden für eine breitere Anwendung des S‑ICD in der klinischen Routine wurden in einem EHRA(European Heart Rhythm Association)-Survey neben den bekannten Limitationen – wie der fehlenden Pacing-Option – v. a. höhere Anschaffungskosten und unzureichende Erstattung genannt [57]. Zukünftige gesundheitsökonomische Analysen sollten klären, ob diese höheren Initialkosten nicht durch geringere Folgekosten, etwa infolge seltenerer Komplikationen und Revisionsoperationen, mehr als ausgeglichen werden.

## Fazit für die Praxis

- Bei geeigneter Selektion bietet der subkutane implantierbare Kardioverter-Defibrillator (S-ICD) einen dem transvenösen ICD (TV-ICD) gleichwertigen Schutz vor dem plötzlichen Herztod, bei deutlich günstigerem Sicherheitsprofil durch weniger prozedurale und elektrodenassoziierte Komplikationen sowie die Vermeidung systemischer Infektionen.
- Bei Bedarf schließt zukünftig ein kommunizierendes, modulares Leadless-Pacing-Modul (Brady-/antitachykardes Pacing) die Stimulationslücke (Zulassungsprüfung laufend).
- Neuere Geräteplattformen und verfeinerte Algorithmen werden die Technologie weiter verbessern.
- Der S‑ICD ist vom Nischenkonzept in die Regelversorgung übergegangen, eine datenbasierte Leitlinienaktualisierung ist absehbar.

## Supplementary Information

Video 1) Präparation einer intermuskulären Tasche durch Eröffnung der intermuskulären Faszie zwischen M. latissimus dorsi und M. serratus anterior. Die Taschenanlage benötigt nur wenige Sekunden, da sie bereits anatomisch vorhanden ist und lediglich die Faszie stumpf eröffnet wird.

Video 2) 1. Tunnelierung: Das Electrode Delivery System (EDS) wird mit einer 11-F-Peel-away-Schleuse vorgeladen und aus der xiphoidalen Inzision streng fasziennah über die Rippen in Richtung Aggregattasche getunnelt; anschließend wird das EDS entfernt, die Schleuse *in situ* belassen und die Elektrode von xiphoidal zur Tasche durch die Schleuse vorgeschoben, danach die Schleuse unter Fixation der Elektrode atraumatisch abgezogen (muss nicht gesplittet werden).

Video 3) 2. Tunnelierung: Das EDS mit 11-F-Peel-away-Schleuse wird aus der xiphoidalen Inzision streng fasziennah parasternal (1–2 cm links des Sternums) nach kranial bis zur markierten Position (ca. 1 cm unterhalb Manubrium, entspricht topographisch in etwa der Carina) vorgeschoben; EDS entfernen, die Schleuse *in situ* belassen und die Elektrode von xiphoidal nach kranial durch die Schleuse vorführen, Coil-Ausrichtung parallel zum Sternum gewährleisten und anschließend die Schleuse aufsplitten und bei fixierter Elektrode atraumatisch abziehen. Dieser Schritt ist am wichtigsten, die Elektrode muss zur Minimierung der Impedanz und besseren Schockeffektivität so sternumnah wie möglich platziert werden. Bei nur 1 cm Abstand von der Coil zum Sternum (z. B. bei adipösen Patienten und ungenauer Tunnelierung) beträgt der PAETORIAN-Score schon 120.

Video 4) Explantation eines S‑ICD und Extraktion der Elektrode nach 6 Jahren. Die Extraktion erfolgt durch einfache manuelle Traktion der Elektrode von der xiphoidalen Inzision und dann durch Zug aus der Tasche. Auch nach vielen Jahren ist die Extraktion innerhalb weniger Sekunden komplikationsfrei durchführbar. Zu sehen ist ferner eine Verkapselung um die Elektrode, die sich gelöst hat bei der Extraktion.
